# COVID-19 vaccinations and their side effects: a scoping systematic review

**DOI:** 10.12688/f1000research.134171.2

**Published:** 2024-09-26

**Authors:** Hind Monadhel, Ayad Abbas, Athraa Mohammed

**Affiliations:** 1 Computer Science, University of Technology-Iraq, Baghdad, 10053, Iraq

**Keywords:** COVID-19 Vaccine, Machine learning, side effects, Statistical methods, Adverse reactions, Data Processing and Analysis, Data Mining.

## Abstract

**Introduction:** The COVID-19 virus has impacted people worldwide, causing significant changes in their lifestyles. Since the emergence of the epidemic, attempts have begun to prepare a vaccine that can eliminate the virus and restore balance to life in the entire world. Over the past two years, countries and specialized companies have competed to obtain a license from the World Health Organization for the vaccines that were discovered. After the appearance of vaccines in the health community, comparisons and fears of their side effects began, but people don’t get an answer to the question of which is the best vaccine.

**Methods:** IEEE Xplore, ScienceDirect, the New England Journal of Medicine, Google Scholar, and PubMed databases were searched for literature on the COVID-19 vaccine and its side effects. we surveyed the literature on the COVID-19 vaccine’s side effects and the sorts of side effects observed after vaccination. Depending on data from the literature, we compared these vaccines in terms of side effects, then we analyzed the gaps and obstacles of previous studies and made proposals to process these gaps in future studies.

**Results:** Overall, 17 studies were included in this scoping systematic review as they fulfilled the criteria specified, the majority of which were cross-sectional and retrospective cross-sectional studies. Most of the side effects were mild, self-limiting, and common. Thus, they usually resolve within 1–3 days after vaccination. Factors associated with higher side effects included advanced age, allergic conditions, those taking other medications (particularly immunosuppressive ones), those with a history of type II diabetes, heart disease, hypertension, COVID-19 infection, and female sex. Our meta‐analyses also found that mRNA vaccines looked to be more effective, while inactivated vaccinations had fewer side effects.

**Conclusion:** This review shows that the COVID-19 vaccine is safe to administer and induces protection.

**Abbreviations**
CDC: Centers for Disease Control and Prevention
COVID-19: Coronavirus Disease 2019
D-dimer: Fibrin degradation product
FDA: Food and Drug Administration
mRNA: messenger ribonucleic acid
VAERS: Vaccine Adverse Event Reporting System
WHO: World Health Organization

## Introduction

COVID-19 is an infectious disease caused by the severe acute respiratory syndrome coronavirus 2 (SARS-CoV-2), which was initially detected in Wuhan, China, in December 2019. As of 18 August 2024, there have been 776 million confirmed cases worldwide, with 3.0 million deaths ( WHO 2024). It belongs to the same group of ribonucleic acid (RNA) viruses that cause Middle East respiratory syndrome (MERS) and severe acute respiratory syndrome (SARS) ( Mahendra 2020). Corona-viruses are a large family of viruses that infect many different species, including humans. In humans, the coronavirus can cause a variety of diseases, from minor respiratory tract infections like the common cold to fatal infections ( Weiss & Navas-Martin 2005).

The virus might be transferred by close touch or simply droplets, between people, when the mucosal membranes of healthy individuals are exposed to secretions generated by the carriers ( Sharma 2020). Symptoms of COVID-19 might vary from person to person. Some of them may exhibit no signs or symptoms of infection, such as breathing difficulties, or they may succumb to the sickness ( Sahin 2020).

Even though several therapeutic compounds and medications have been proposed and reused in the battle against COVID-19, they have remained supportive therapy choices; however, vaccines remain the most efficient and effective way to protect people from this disease ( Tavilani et al. 2021).

The development of vaccinations has made significant advances; the first mass vaccination program started in early December 2020 in Europe, with priority given to individuals who are at high risk of severe COVID-19 infection, such as the elderly, and those who are at high risk of viral exposure and transmission, such as front-line medical staff ( Paules et al. 2020). 7,952,750,402 vaccine doses have been provided globally through December 6, 2021 ( WHO 2021c).

However, no vaccine is entirely free from complications or adverse reactions. Any vaccination can have early adverse reactions, including local ones like pain, swelling, and redness, as well as systemic ones like headache, chills, nausea, fatigue, myalgia, and fever ( Al Khames et al. 2021). Following the SARS-CoV-2 vaccination, there have been several recorded occurrences of side effects; in addition, there have been a small number of deaths ( Varsavsky 2021). This has been recorded with COVID-19 vaccinations, and it has been observed in people with pre-existing health conditions like diabetes and high blood pressure, as well as people who have had allergic reactions to the vaccine. The extent to which they are linked to vaccines, however, is unknown and under investigation ( Hussien 2019).

In this systematic review, we aim to make reference to the wide range of possible reactions that can occur after vaccinations, so that healthcare providers can better understand these patients. The purpose of this scoping systematic review is to summarize and integrate the results of studies on the side effects of the COVID-19 vaccination and then determine the gaps in these studies. This paper is organized in the following manner: Section 1 is an introduction to COVID-19 viruses and vaccination, Section 2 is Background, section 3 is the methods, Section 4 is a review of the Conducted Studies, Section 5 the results, section 6 discusses the results & gaps in the literature studies, section 7 concludes the most important points of this paper and section 8 represent study strengths and limitations.

### Review question

What are the rates of side effects of COVID-19 vaccines, and which symptoms are the most common?

## Background

### A brief overview of the vaccine

A vaccine is a biologics that provides Active-Adaptive immunity against a particular disease. In vaccination development, the micro-organisms that cause the disease are utilized in either attenuated or killed form or involve using their toxins or surface proteins. in order to activate the immunological system against foreign bodies, Vaccines are administered nasally, orally, or by injection (intramuscularly, subcutaneously, or intradermally) ( Dai et al. 2019). To develop immunity, the body produces antibodies (immunoglobulins) against microorganisms, thus generating the body’s defense mechanism. The antibodies produced by the immune system in response to re-exposure to the same microorganism prevent or lower disease severity ( WHO 2020; Dai et al. 2019).

### Cause of side effects

Any unwanted and unintended or harmful reactions following the administration of a medicine, vaccine, or combination of vaccinations are known as adverse reactions. It may be a known adverse event or previously unrecognized ( Salisbury et al. 2006). The COVID-19 vaccinations contain genetic blueprints for producing spike (S) proteins that sit on the coronavirus’s surface and allow it to enter human cells. Upon receiving these instructions, human cells produce copies of the spike (S) protein. In fact, since cells only produce a portion of the virus and not the whole pathogen, we are not infected. While foreign spikes cannot cause disease, they can activate the immune system exactly as it is supposed to do. As a result of the strong immune response triggered by vaccines, a wide range of symptoms can be triggered, including fever, fatigue, chills, inflammation, and reactions at the injection site. The innate immune system response to the COVID-19 vaccine causes the reactions ( Marsa 2021).

### COVID-19 Vaccines: Types and mechanism of action

***Messenger RNA***

These vaccines involve using synthetically manufactured Messenger RNA (mRNA), that infects host cells and produces a component of the spike protein. After the body degrades it, the protein triggers the production of antibodies. These immunoglobulins (antibodies) prepare the body to deal with any infection in the future with a minimal risk of side effects. This mechanism is used by the Pfizer and Moderna vaccines ( CDC 2021a). The BNT162b2 developed by Pfizer/BioNTech produces an immunological response by eliciting IgG, IgA, CD8+ cells, or CD4+ cells, whereas mRNA-1273 developed by Moderna stimulates CD8 T cells ( Goyal et al. 2021).

***Viral vector-based vaccines***

They are altered versions of a virus belonging to a different genus that is utilized as a vector. Through its interaction with immune cells, it helps them recognize and outwit pathogenic viruses. The immune cells of the body recognize the presence of foreign antigens as soon as they are injected into the body, and activate an immune response by releasing antibody-producing B cells and T cells that search down infected cells and destroy them. T cells function by examining the storage of proteins that are expressed on the cell’s surfaces. When they encounter a foreign protein, they trigger an immune response against the cell storing it because they are able to identify the body’s own proteins as “self” ( WHO 2021a). This mechanism is used by Janssen/Johnson & Johnson, Sputnik V, and AstraZeneca vaccines ( CDC 2021b).

***Whole virus or inactivated virus vaccines***

An age-old and common vaccine, there are currently two types: live-attenuated and inactivated. Chemicals, heat, and radiation are used to destroy the virus’s genetic material in order to prepare inactivated vaccinations. Because these vaccines are versions of weak natural pathogens, the immune system activates a wide range of defense mechanisms in response, including killer T cells that destroy the infected cells, helper T cells that help produce antibodies, and antibodies-producing B cells that attack pathogens ( WHO 2021b). When they are introduced into the body, they induce antibody-mediated responses that are weak and relatively short-lived. Therefore, they are administered along with an adjuvant, and often booster doses are required. The live attenuated vaccines, in contrast, use a weakened version of the virus inside the body. These viruses can grow and reproduce once they are inside the body, yet they do not cause symptomatic disease in the host ( NIAID 2021). This inactivated mechanism is used by Sinopharm, Covaxin, Sinovac, and Corovac vaccines. The live attenuated mechanism is used in the Covivac vaccine ( Shahzamani et al. 2021).

Table 1 depicts a comparison of the COVID-19 vaccines that were included in the research studies; moreover.

**Table 1.** A comparison of the COVID-19 vaccines ( Vaccine Characteristics, 2023; COVID-19 vaccine, 2023; Covaxin COVID-19 vaccine: What to know about side effects, 2023; Tavilani et al. 2021).

	Moderna	Pfizer-BioNTech	AstraZeneca	Sinopharm	Bharat Biotech
Type of vaccine	messenger ribonucleic acids (mRNA) vaccine	messenger ribonucleic acids (mRNA) vaccine	Adenovirus Viral vector vaccine	Inactivated Virus Vaccines	Inactivated Virus Vaccines
Other names	mRNA-1273	BNT162b2, Comirnaty	AZD1222 (ChAdOx1)	BBIP-CorV	Covaxin (BBV152)
Manufactured in	Spain	Germany	Sweden	China	India
Approval	World Health Organization (WHO), Food and Drug Administration (FDA), European Medicines Agency (EMA).	World Health Organization (WHO), Food and Drug Administration (FDA), European Medicines Agency (EMA).	World Health Organization (WHO), European Medicines Agency (EMA).	World Health Organization (WHO), Food and Drug Administration (FDA), European Medicines Agency (EMA).	World Health Organization (WHO),
Age groups	Adults (FDA), 6 Years and up (EMA)	5 Years and up	18 Years and up	18 Years and up	18 Years and up
Storage	stored for 30 days between 2°C and 8°C.	stored for 6 months at − 70 °C. Undiluted vials can be stored at room temperature for no more than 2 h.	Store in a refrigerator (2–8 °C). Do not freeze. Preserve the vials from light.	Store in a refrigerator between 2 °C and 8 °C.	Stored between 2°C and 8°C for up to 6 hours.
Number of doses	2 shots, given 28 days apart.	2 shots, given 21 days apart.	2 shots, given 28 days apart	2 shots, given 21 days apart	2 shots, given 28 days apart
Effectiveness	94.5% in preventing COVID-19 infection.	95% in preventing COVID-19 infection.	72% in preventing COVID-19 infection.	79% in preventing COVID-19 infection.	78% in preventing COVID-19 infection.
When you shouldn’t get the vaccine	1. Have a severe allergic reaction after a previous dose of the vaccine. 2. Individuals who developed myocarditis or pericarditis following the first dose of vaccine. 3. Have a severe allergic reaction to any of the ingredients.	1. Have a severe allergic reaction to any of the ingredients. 2. Anyone with a fever (body temperature over 38.5°C).	1. Have a severe allergic reaction to any of the ingredients. 2. Individuals who have had Thrombosis with thrombocytopenia (TTS) after the first dose.	1. Have anaphylaxis to any of the ingredients. 2. Anyone with a fever (body temperature over 38.5°C).	1. Have anaphylaxis to any of the ingredients. 2. Cases with acute PCR-confirmed COVID-19. 3. Anyone with a fever (body temperature over 38.5°C). 4. People with bleeding disorders.
Common side effects	1. Chills 2. Tiredness 3. Headache 4. Pain (at the injection site) 5. Swelling 6. Readiness (at the injection site). Other side effects listed in WHO: are muscle and joint pain, vomiting, fever, and a remote chance of severe allergic reactions.	1. Chills 2. Tiredness 3. Headache 4. Pain (at the injection site) 5. Swelling 6. Readiness (at the injection site). Other side effects listed in WHO: are muscle and joint pain, feeling unwell, swollen lymph nodes, non-severe allergic reactions, and severe allergic reactions.	1. Tiredness 2. Headache 3. Nausea 4. Pain, Readiness, Bruising, or Warmth at the injection site 5. Vomiting 6. Chills Other side effects listed by WHO: are joint pain, fever, diarrhea, muscle ache, and a remote chance of Guillain-Barre syndrome (GBS).	1. Pain, Swelling, and Readiness at the injection site. 2. Fatigue 3. Headache 4. Fever or Chills	1. Pain, Swelling, or both at the injection site. 2. Fever 3. Irritability 4. Headache Other side effects listed in WHO: a remote chance of severe allergic reactions.
Can this vaccine be ‘Mixed and matched ‘with other Vaccinations	Mixing COVID-19 vaccines is only allowed for booster shots. 1. Based on the availability of the product, either of the WHO EUL COVID-19 vectored vaccinations (Janssen or AstraZeneca Vaxzervria/COVISHIELD) may be used as a second dose after a **first dose** of the Moderna vaccine. 2. A **second dose** of the Moderna vaccination may be given following any of the WHO COVID-19 inactivated vaccinations (Sinopharm, Sinovac, Bharat) or vectored vaccinations (Janssen Vaxzervria/COVISHIELD). 3. A booster dose of Moderna can be given after any other COVID-19 vaccine.	Mixing COVID-19 vaccines is only allowed for booster shots. 1. Based on the availability of the product, either of the WHO EUL COVID-19 vectored vaccinations (Janssen or AstraZeneca Vaxzervria/COVISHIELD) may be used as a second dose after a **first dose** of the Pfizer vaccine. 2. A **second dose** of the Pfizer vaccination may be given following any of the WHO COVID-19 inactivated vaccinations (Sinopharm, Sinovac, Bharat) or vectored vaccinations (Janssen Vaxzervria/COVISHIELD). 3. A booster dose of Pfizer can be given after any other COVID-19 vaccine.	Mixing COVID-19 vaccines is only allowed for booster shots. 1. Based on the availability of the product, either of the mRNA COVID-19 vaccines (Pfizer or Moderna) may be used as a second dose after a **first dose** of the AstraZeneca vaccine. 2. A **second dose** of the Astra-Zeneca vaccination may be given following any of the WHO COVID-19-inactivated vaccinations (Sinopharm, Sinovac, or Bharat).	Mixing COVID-19 vaccines is only allowed for booster shots. 1. Based on the availability of the product, Either of the WHO EUL COVID-19 mRNA vaccines (Pfizer or Moderna) or vectored vaccines (AstraZeneca Vaxzervria/COVISHIELD or Janssen) may be used as a second dose after a **first dose** of the Sinopharm vaccine.	Mixing COVID-19 vaccines is only allowed for booster shots. 1. Based on the availability of the product, Either of the WHO EUL COVID-19 mRNA vaccines (Pfizer or Moderna) or vectored vaccines (AstraZeneca Vaxzervria/COVISHIELD or Janssen) may be used as a second dose after a **first dose** of the Bharat vaccine.

## Methods

The PRISMA guidelines for systematic reviews and meta-analyses were followed in this research ( Moher et al. & PRISMA Group*. 2009). A structured and transparent approach was implemented to identify, select, and synthesize relevant studies on the side effects of COVID-19 vaccines.

### Eligibility criteria

**Inclusion criteria**

Studies were included in the review if they specifically examined COVID-19 vaccines and reported on any side effects or adverse reactions following vaccination. Only peer-reviewed articles that provided comprehensive data on the safety and efficacy of vaccines were considered.

**Exclusion criteria**

The following types of studies were excluded from the review:

• Abstracts, preprints, and grey literature, as these often lack the peer-review process and comprehensive data required for rigorous analysis.
• Book chapters, non-English studies, case reports, and studies unrelated to the topic were also excluded to maintain focus on peer-reviewed, original research directly relevant to the review’s objectives.

### Screening process

Records were meticulously screened by reviewers to ensure studies met inclusion criteria, with titles and abstracts assessed initially. Discrepancies were resolved collaboratively, and full-text articles were retrieved for potentially eligible studies. Ayad Abbas and Athraa Mohammed supervised and reviewed, while Hind Monadhel handled analysis, resources, visualization, draft preparation, and editing.

## Information sources

We searched through the databases of IEEE Xplore, ScienceDirect, the New England Journal of Medicine, Google Scholar, and PubMed to find studies about COVID-19 and the side effects of COVID-19 vaccinations. from 2019 to 2022 to select eligible research publications. The searches were performed without time filters, and backward snowballing was used to find articles that weren’t found in the initial search by screening relevant studies and their references ( Figure 4). These databases had been selected based on several collected journals & conferences about emerging topics, including the Coronavirus disease (COVID-19) and the side effects related to the COVID-19 vaccine.

## Search strategy

Our keyword search will be based on the following combination of Medical Subject Headings: (COVID-19 OR SARS-CoV-2 OR 2019-nCoV OR coronavirus OR MERS-CoV OR SARS-CoV) AND (Vaccines OR Vaccination OR COVID-19 vaccine OR SARS-CoV-2 vaccine OR MERS-CoV vaccine OR mRNA vaccine OR Moderna vaccine OR Pfizer-BioNTech COVID-19 vaccine OR mRNA-1273 vaccine OR ChAdOx1covid-19 vaccine OR AZ1222 covid-19 vaccine OR Johnson & Johnson covid-19 vaccination OR Ad26 covid-19 vaccine OR BIBP-CorV vaccine OR BIBP vaccine OR CDC-approved COVID-19 vaccines OR Sinopharm vaccine OR AstraZeneca-Oxford vaccine OR BNT162b2 COVID-19 Vaccine) AND (Toxicity OR harmful impact OR side effects OR adverse events OR negative reactions) as shown in Table 2.

**Table 2.** Search strategy.

Search items	The term
**1**	COVID-19
**2**	SARS-CoV-2
**3**	2019-nCoV
**4**	coronavirus
**5**	MERS-CoV
**6**	SARS-CoV
**7**	Vaccines
**8**	Vaccination
**9**	COVID-19 vaccine
**10**	SARS-CoV-2 vaccine
**11**	MERS-CoV vaccine
**12**	mRNA vaccine
**13**	Moderna vaccine
**14**	Pfizer-BioNTech COVID-19 vaccine
**15**	mRNA-1273 vaccine
**16**	ChAdOx1covid-19 vaccine
**17**	AZ1222 covid-19 vaccine
**18**	Johnson & Johnson covid-19 vaccination
**19**	Ad26 covid-19 vaccine
**20**	BIBP-CorV vaccine
**21**	BIBP vaccine
**22**	OR CDC-approved COVID-19 vaccines
**23**	Sinopharm vaccine
**24**	AstraZeneca-Oxford vaccine
**25**	BNT162b2 COVID-19 Vaccine
**26**	Toxicity
**27**	Harmful impact
**28**	Side effects
**29**	Adverse events
**30**	Negative reactions

## Classification of research articles

**Figure 1.**
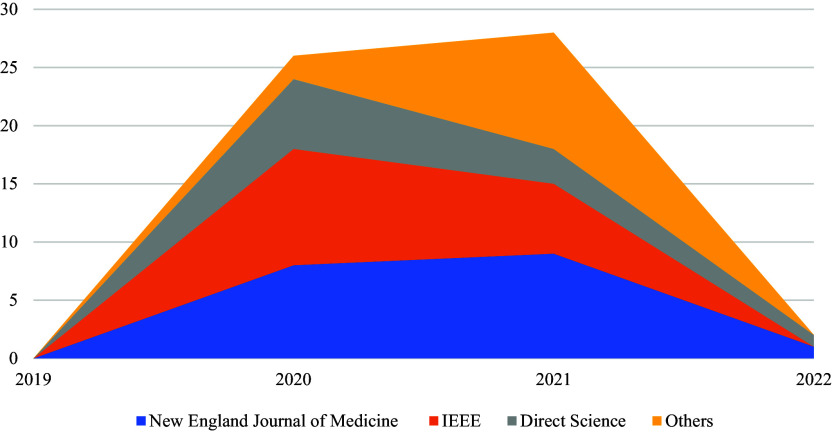
Trends in publication among the five databases.

This section classifies articles according to criteria such as the year of publication and publishing house or journal. Figure 1 below shows the trend of article publications in various databases (i.e., IEEE Xplore, ScienceDirect, the New England Journal of Medicine, Google Scholar, and PubMed) from 2019 to 2022. Annual increases in publication rates are observed. Therefore, COVID-19 vaccines have in recent years gained increasing interest among researchers. Thus, further studies are expected to be conducted in the future.

**Figure 2.**
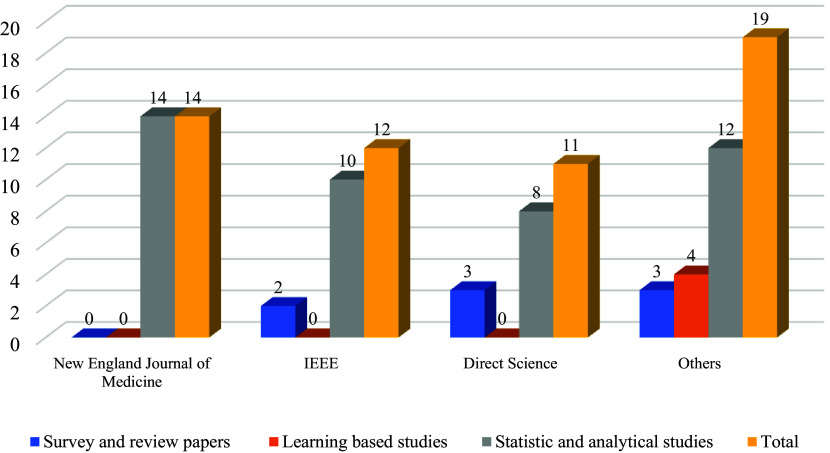
Articles in the different categories of published journals.

Figure 2 illustrates how research articles are distributed based on their orientation: survey or review papers, learning-based studies, and statistical and analytical studies. It is noticeable that a large portion of the papers is statistical since our paper attempts to investigate the side effects of vaccines. In the systematic literature review, there are three categories of research studies: survey and review studies, studies that apply machine learning, and studies that use statistics and analytical methods. The New England Journal of Medicine has published 14 articles in the statistics and analytical methods category. IEEE has published 12 articles. Two of them are survey and review studies, and the other 10 are about statistics and analytical methods. In ScienceDirect 11 articles have been published as follows: survey and review studies (3), statistics & analytical studies (8). In Google Scholar and PubMed, 19 articles have been published as follows: survey and review studies (3), machine learning studies (4), and statistics and analytical studies (12).

## Review of the conducted studies

To gain a better understanding of COVID-19 vaccines and predict and analyze their side effects, several studies were conducted between 2020 and 2021. We highlight a few of these studies in this section, which presents the categories of relevant research papers based on their classification (see Figure 4).

**Figure 3.**
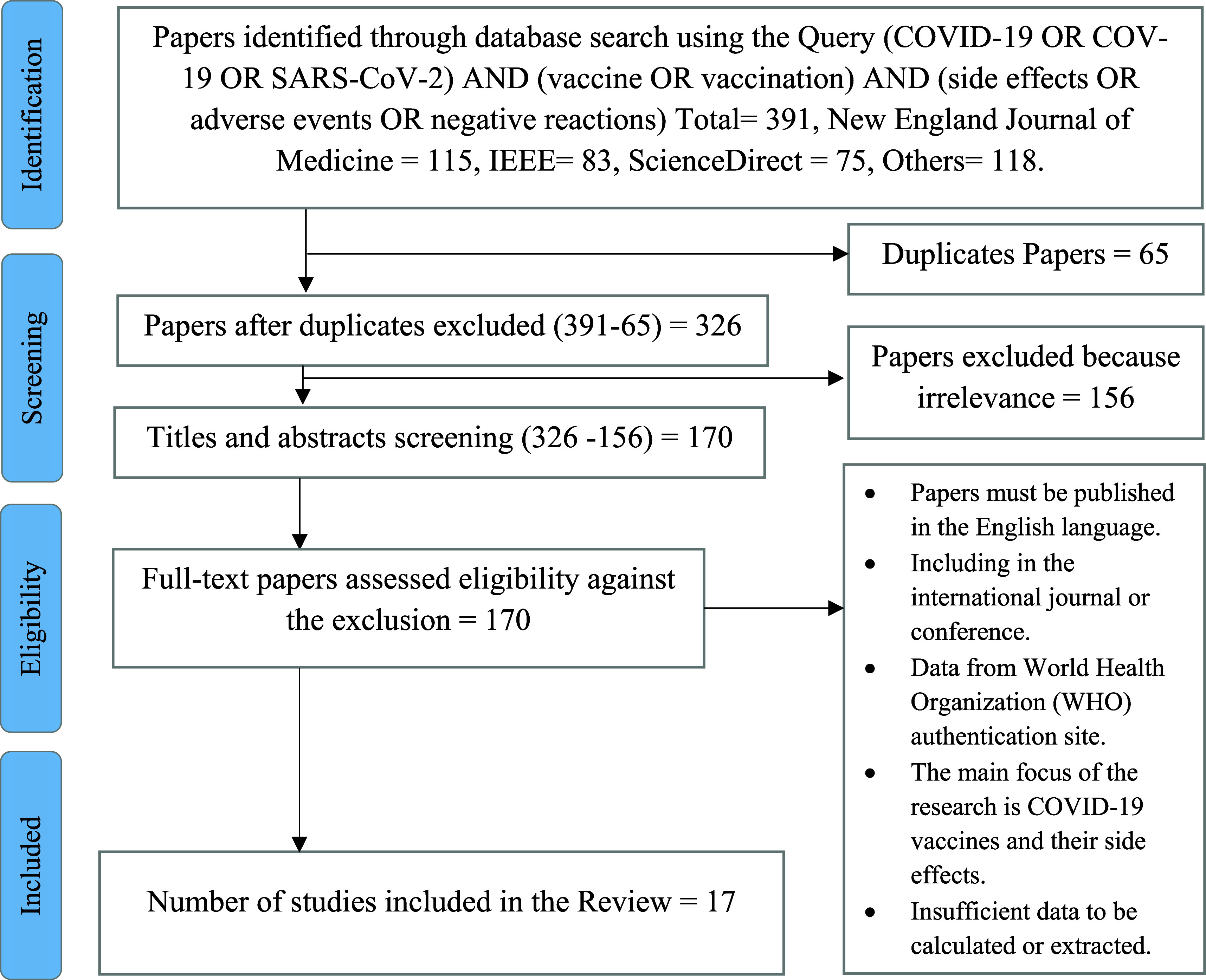
Flow diagram of the search for eligible studies on the side effects, safety, and toxicity of the COVID-19 vaccine.

**Figure 4.**
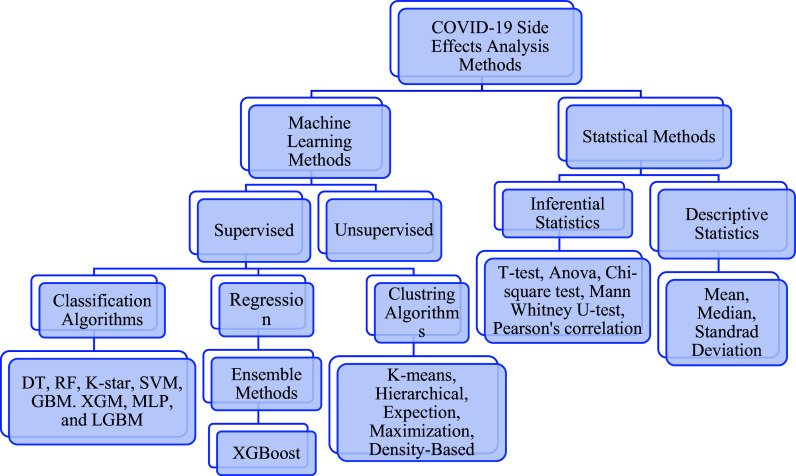
Methods of determining the side effects of COVID-19 Vaccines.

**Prediction of the COVID-19 vaccine that is associated with a higher percentage of side effects using machine learning methods** In ( Abdulrahman et al. 2021) demonstrate how the clustering techniques utilized in the instance of COVID-19 vaccine side effects datasets help to give accuracy for clustering the COVID-19 vaccine side effects datasets. Using the WEKA tool, this paper compared four clustering techniques. It also describes the datasets in terms of multiple-precision factors, cluster case, number of iterations, and time, the findings of these algorithms, the clustering methods employed, and their accuracy. The clustering algorithm k-means was discovered to be frequently used in several types of COVID-19 vaccine side effects datasets with great accuracy.

**Prediction of side effects associated with the COVID-19 vaccines and their severity using machine learning methods** In ( Ahamad 2020) the authors aim to categorize and define patients at risk of such responses using patient data and discover probable common reasons for such side effects to find treatments that lower patient risk. They examined patient medical records and data on the impacts and consequences of immunization. The data was analyzed using a collection of machine learning (ML) classification methods. In most cases, similar traits were shown to be closely connected to negative patient reactions. They included prior infections, hospitalizations, and SARS-CoV-2 reinfection. Medical history-based ML classifiers also could determine which patients were most likely to receive a vaccination with no side effects reliably. In ( Hatmal et al. 2021) the authors evaluated the side effects and perceptions of the COVID-19 vaccines in Jordan. An online poll was distributed to Jordanians who had received any COVID-19 vaccines as part of a cross-sectional investigation. Data were statistically examined, and machine learning (ML) algorithms were utilized to predict the severity of side effects. Despite significant differences in the presence and severity of side effects among these vaccines, statistical analysis revealed that they provide comparable protection against COVID-19 infection. The current paper confirmed that the CDC-approved COVID-19 vaccines are safe and that vaccination makes people feel safer. However, severe cases may necessitate additional medical care or perhaps hospitalization.

Table 3 compares the results of studies based on machine learning techniques about the COVID-19 side effects. Evaluation of Machine Learning Models Eq. (1) was used to evaluate ML models by calculating their accuracies ( Grandini et al. 2020).

$$\text{Accuracy}=\frac{\mathrm{TP}+\mathrm{TN}}{\mathrm{TP}+\mathrm{TN}+\mathrm{FB}+\mathrm{FN}}$$ (1)

**Table 3.** Comparison of COVID-19 vaccines side effects based on ML.

Ref. No.	Population	Vaccine name	Accuracy
Abdulrahman et al. (2021)	3685	Pfizer-BioNTech	67%
Ahamad (2020)	2405	Pfizer, BioNTech, and the mRNA	89%
Hatmal et al. (2021)	2213	Sinopharm, AstraZeneca, Pfizer-BioNTech, and other vaccines	80%

**A variety of statistical methods have been used to assess the side effects after vaccinations** ( Jayadevan 2021) aim to evaluate the initial reaction to the COVID-19 vaccine’s first dosage and to investigate the range of post-vaccination symptom profiles for different vaccinations. A cross-sectional online survey was conducted in India that included questions about the immediate post-vaccination experience. Over a week, from January 29 to February 4, 5396 persons replied to the poll. Overall, 65.9% of respondents said they had had one post-vaccination symptom. 79% of those who reported symptoms did so within the first 12 hours. 472 people reported COVID-19 out of 5396 (or 8.7%). Their symptom profile was similar to those who had no prior experience. In ( El-Shitany 2021) the authors obtained their data using a Google Form questionnaire (online survey). The results were based on the responses of 455 people, all of whom live in Saudi Arabia. After the first and second doses, the vaccine’s side effects were documented. The most common complaints were injection site pain, headaches, flu-like symptoms, fever, and tiredness. Fast heartbeat, generalized aches, difficulty breathing, joint discomfort, cold, and exhaustion whereas some of the less common side effects. Lymph node enlargement and pain, as well as Bell’s palsy, are uncommon side effects. Recipients who had previously been infected reported more difficulty breathing than those who had never been infected with the coronavirus.

Zahid (2021) looked into the most common side effects associated with these four vaccines’ first and second doses. 311 people who had two doses of one of these four vaccinations completed an online questionnaire, which was used to collect data. The findings of this paper demonstrated that the second dose of the vaccine caused higher side effects, independent of the vaccine’s identification. Overall, vaccination recipients (Sinopharm) experienced the fewest side effects among the four vaccines examined. The most important conclusion of the paper is that the post-vaccination side effects of the first and second doses were modest and predictable, with no hospitalizations; this information can help reduce vaccine fear.

**As for comparing the side effects of the COVID-19 vaccines** ( Andrzejczak-Grządko et al. 2021) compared the AstraZeneca and Pfizer vaccines’ side effects. The survey results suggest that side effects from the AstraZeneca vaccine’s first dose are more common than those from the Pfizer vaccine’s first and second doses. In this survey, most respondents reported at least one side effect after receiving the AstraZeneca and Pfizer vaccines, but these reactions were less common after receiving the Pfizer vaccine. A survey was made available on the internet. It was done on patients who had been immunized with Pfizer or AstraZeneca vaccines. The respondents were asked about the side effects of the vaccines after the first and second doses. In ( Almufty et al. 2021) the authors highlighted probable side effects from covid19 vaccines, a comparison of the three COVID-19 vaccines now available in Iraq, and risk factors for severe side effects (Sinopharm, AstraZeneca-Oxford, and Pfizer- BioNTech). A standardized questionnaire platform was used to collect information about the Iraqi people. The majority of the symptoms ranged from mild to moderate in severity. With P values less than 0.0001, both residencies in Iraq’s Kurdistan Region and the AstraZeneca vaccine were statistically significant risk factors for experiencing severe symptoms.

**In regards to young age groups and side effects** Alamer et al. (2021) looked into the early side effects of the Pfizer-BioNTech (BNT162b2) mRNA vaccine in Saudi Arabian youngsters aged 12 to 18. A cross-sectional study using a self-administered online survey was done to examine the side effects in children in this age range after receiving one or two doses of the Pfizer-BioNTech (BNT162b2) mRNA vaccine. After vaccination, general and demographic information was acquired, and vaccine-related side effects were examined.

**As the safety assessment of the COVID-19 vaccines is necessary,** in Dziedzic et al. (2021). The goal was to see if the ChAdOx1 n-COV-19 vaccine was safe in terms of systemic and local side effects on patients 48 hours after injection. This paper report has three (3) particular aims. First, the paper report assesses and analyzes the safety of the first and second doses of the ChAdOx1 n-COV-19 vaccinations 48 hours after administration. Second, the paper examines the side effects of the ChAdOx1 n-COV-19 vaccination among people younger than 40 years and people older than 40 years following delivery over a two-day (48-hour) period to evaluate and compare the vaccine’s safety by age and sex.

**To analyze the side effects from different aspects** in Azimi et al. (2021), the authors aimed to determine the frequency, intensity, and degree of side effects linked with the COVID-19 vaccination (ChAdOx1 nCoV-19 vaccine or AstraZeneca) among Kabul University of Medical Sciences employees. A retrospective observational, interview-based study was undertaken among the Kabul University of Medical Science’s faculty and instructors in Kabul, Afghanistan, from April 4 to April 20, 2021, to examine the COVID-19 vaccine’s side responses (ChAdOx1 nCoV-19 vaccine or AstraZeneca). Participants were questioned after the first AstraZeneca vaccination dosage was given out. They were instructed to report any negative reactions to the vaccination within 8–10 days of getting it. Klugar et al. (2021) aimed to assess the post-vaccination side effects of the various vaccines approved in Germany. An online questionnaire that has been validated and evaluated for a priori dependability was used in a cross-sectional survey. Demographic information, medical and COVID-19-related anamneses, and local, systemic, oral, and skin-related side effects following COVID-19 immunization were all asked about in the survey. Additionally, ( Riad et al. 2021) conducted a cross-sectional survey-based study in the Czech Republic to gather data on the COVID-19 vaccine’s side effects among healthcare workers. The researchers used a validated questionnaire containing twenty-eight multiple-choice items about demographics, medical anamneses, COVID-19-related, and general, oral, and skin-related side effects. The most often used drugs with side effects were antihistamines, demanding a greater investigation. Overall, people who took two doses experienced more side effects. The manufacturer’s figures matched the distribution of side effects among Czech healthcare personnel, especially their link with younger age groups and the second dosage.

Also, Kitagawa et al. (2022) conducted an online survey of people who had self-reported side effects after receiving two doses of BNT162b2 or mRNA-1273 immunization. The incidence of adverse effects was investigated after each dose of vaccination. The incidence of side effects following the second dose of the BNT162b2 and mRNA-1273 vaccines was compared using propensity score matching.

**To examine the duration of side effects’ appearances,** a study conducted by Alhazmi et al. (2021) aimed to see how long it took for post-vaccination adverse effects to appear. An online questionnaire was used to perform cross-sectional, retrospective research among COVID-19 immunization participants in Saudi Arabia. General and demographic data were collected after at least one immunization dose, and vaccine-related side effects were examined. The side effects described by their paper participants after receiving the Oxford-AstraZeneca and Pfizer-BioNTech vaccines are similar to those reported in clinical trials, showing that both vaccines have safe profiles.

**In terms of reducing vaccine hesitancy** Saeed et al. (2021) aimed to gather data on Sinopharm’s COVID-19 vaccine’s side effects. Between January and April 2021, a cross-sectional survey paper was done to gather data on the effects of the COVID-19 vaccine among UAE residents. The response of persons who refused to take the COVID-19 vaccine was reported, as demographic data, vaccination, and the reasons people did not want to take the COVID-19 vaccine. The post-vaccination side effects of the first and second doses were modest and predictable, with no hospitalizations; this knowledge can assist in reducing vaccine fear. Moreover, Kadali et al. (2021) looked at the side effects of the mRNA 1273 vaccine by going over all of the organ systems in depth an impartial online survey tool was used in a randomized, cross-sectional investigation to collect responses from HCWs. The bulk of the symptoms did not appear to be life-threatening. This vaccination appears to have a better level of acceptability, despite the wide variety of self-reported effects.

## Results

**Figure 5.**
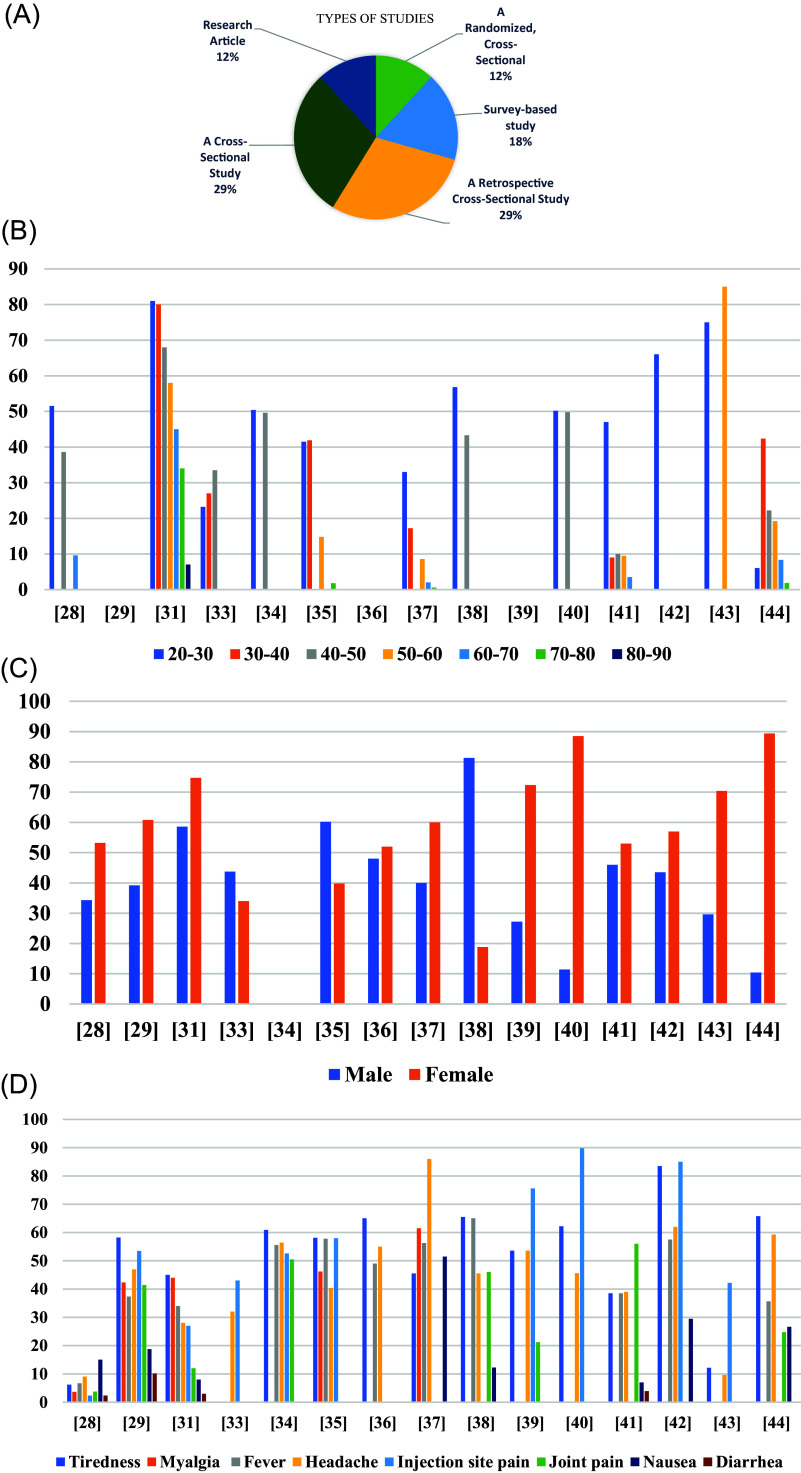
A: The types of research studies that were evaluated. B: The incidence and severity of post-vaccination symptoms with age. C: Post-vaccination symptoms among males vs. females. D: Comparison of the side effects and it can be seen that injection site pain ranked highest in side effects, followed by tiredness.

According to the search strategy, 391 papers were identified. A total of 170 papers were screened for eligibility by full-text review after titles and abstracts were screened. Following a thorough full-text screening, 114 papers were deemed unacceptable for the reasons listed in ( Figure 3). The remaining 17 papers ( Table 5) were included in this scoping systematic review as they fulfilled the criteria specified. The majority of studies were designed as randomized cross-sectional studies (n = 2), survey-based studies (n = 3), retrospective cross-sectional studies (n = 5), cross-sectional studies (n = 5), and research article studies (n = 2) ( Figure 5A).

The final analysis of the studies, included 38680 individuals who experienced at least 1 or more side effects were involved from twelve countries representing three regions of the world, representing as North America (n = 1), South & Western Asia (n = 9), and Central Eastern European region (n=2). A detailed description of the studies ( Abdulrahman et al. 2021; Ahamad, 2020; Ma’mon, 2021; Grandini et al. 2020; Jayadevan, 2021; El-Shitany, 2021; Zahid, 2021; Andrzejczak-Grządko et al. 2021; Almufty et al. 2021; Alamer et al. 2021; Dziedzic et al. 2021; Azimi et al. 2021; Klugar et al. 2021; Riad et al. 2021; Kitagawa et al. 2022; Alhazmi et al. 2021; Saeed et al. 2021; Kadali et al. 2021) is provided in Table 5. Among the Vaccine platforms included in the systematic review were DNA (2), inactivated (2), and mRNA (2). There were seven (7) studies in which healthcare workers were the study population, while twelve (12) studies featured a majority of female participants. Among the cases reported, most side effects were reported in women and age groups between 20 and 30 ( Figure 5B & C). The max number of participants in Kitagawa’s study was around 12109 and the min number of participants in Dziedzic’s study was only 247 participants. Participants in the studies were all adults and adolescents aged 18 or older. Except for Alamer’s study, participants were between 12 and 18 years old ( Table 5).

### Local and systemic side effects

Local side effects were more prevalent than systemic side effects, and the reported side effects were significantly higher among females than males. Most of participants, however, experienced mild and short-term side effects. Severe reactions or anaphylactic reactions may occur in rare cases. The most reported local and systemic side effects for the vaccines were pain at the site of the injection and tiredness, respectively ( Figure 5D). Adenovirus Viral vector COVID-19 vaccines had a higher incidence of side effects than other COVID-19 vaccines in most of the studies. Max side effects of injection site pain reported in Riad’s study around 89.8%. Max side effects of Tiredness reported in Alhazmi’s study was around 83.5% ( Table 4). It was reported in half of the studies that side effects lasted between 1-3 days.

**Table 4.** Post-vaccination symptoms percentage of COVID-19 vaccine based on a statistical approach.

Ref. No.	Tiredness	Myalgia	Fever	Headache	Injection site pain	Joint pain	Nausea	Diarrhea
Jayadevan (2021)	45%	44%	34%	28%	27%	12%	8%	3%
Zahid (2021)	-	-	-	32%	43%	-	-	-
Andrzejczak-Grządko et al. (2021)	60.9%	-	55.6%	56.4%	52.6%	50.5 %	14/2.7%	-
Almufty et al. (2021)	58.1%	46.2%	57.8%	40.4%	58%	-	-	-
Alamer et al. (2021)	65%	-	49%	55%	-	-	-	-
Dziedzic et al. (2021)	45.8%	61.5%	56.3%	86%	-	-	51.5%	-
Azimi et al. (2021)	65.5%	-	65%	45.5%	-	46%	12.5%	-
Klugar et al. (2021)	53.6%	-	-	53.6%	75.6%	21.2%	-	-
Riad et al. (2021)	62.2%	-	-	45.6%	89.8%	-	-	-
Kitagawa et al. (2022)	38.5%	-	38.5%	39%	-	56%	7%	3.9
Alhazmi et al. (2021)	83.5%	-	57.5%	62%	85%	-	29.5%	-
Saeed et al. (2021)	12.2%	-	-	9.6%	42.2%	-	-	-
Hatmal et al. (2021)	58.20%	42.34%	37.37%	46.99%	53.45%	-	18.79%	10.16%
Kadali et al. (2021)	65.74%	-	35.65%	59.26%	-	24.77%	26.62%	-

### Oral and skin side effects

**Figure 6.**
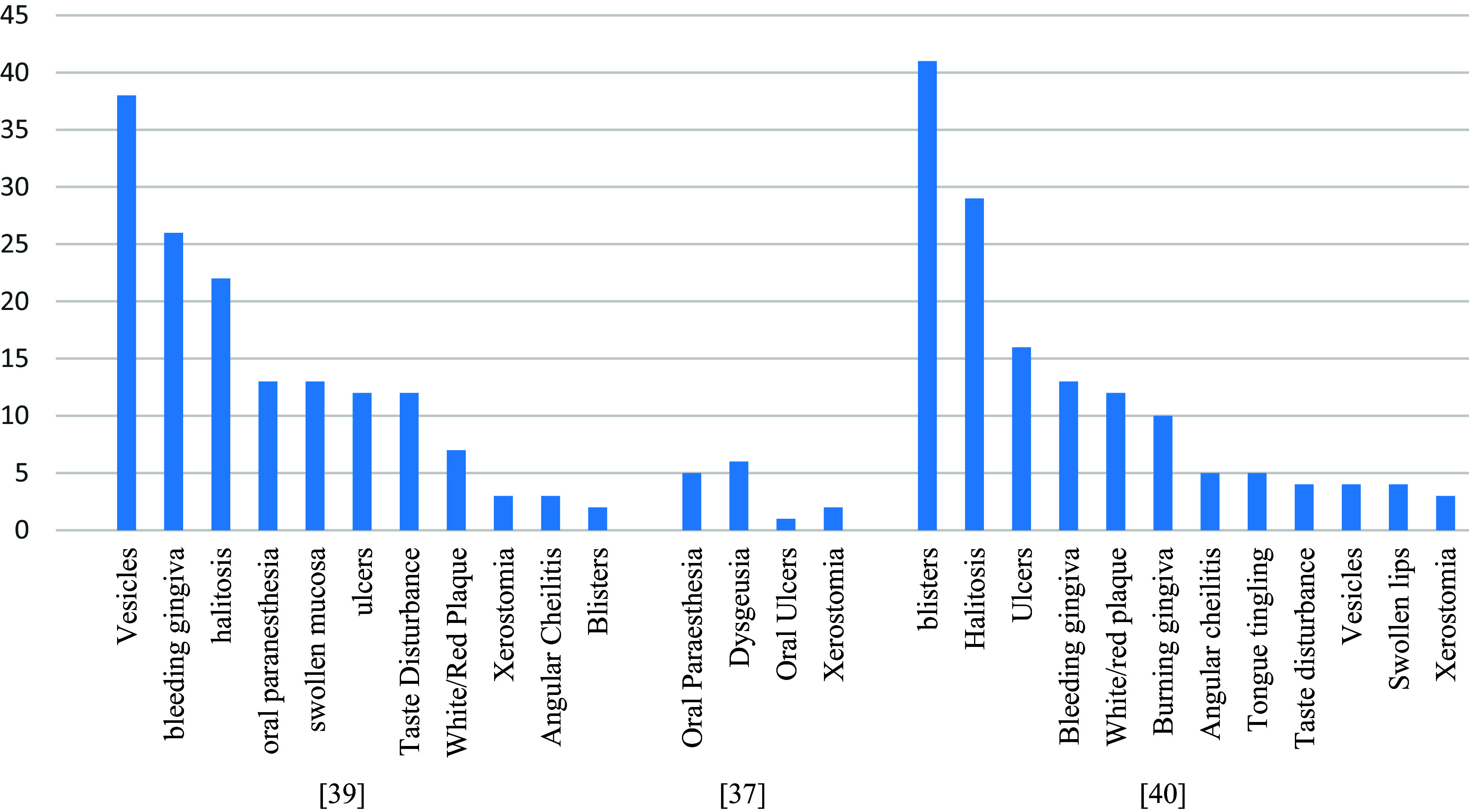
The most commonly reported oral side effects.

**Figure 7.**
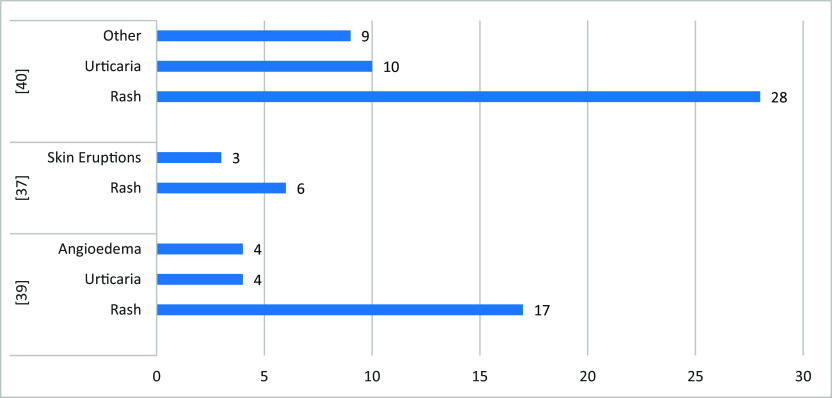
The most commonly reported skin-related side effects.

A total of 533 cases of oral and skin side effects have been reported in three studies following vaccination with COVID-19. The most common oral side effects were vesicles (6.3%), bleeding gingiva (4.3%), oral paraesthesia (2.2%), dysgeusia (2.4%), blisters (36%), and halitosis (25.4%) ( Figure 6). While rash and urticaria (other than at the site of the injection) were the most reported skin-related side effects ( Figure 7). Three-fourths (75.1%) of the oral side effects appeared within the first week following vaccination. The most affected sites for skin-related side effects were the upper limb, face, chest (trunk), lower limb, and back.

### Factors associated with side effects of COVID-19 vaccines

The association between patients’ medical histories and side effects was only looked at in some studies ( Ahamad 2020).

According to the results, patient medical histories are strongly correlated with the incidence of side effects, some of which can be severe and even fatal. Patients with advanced age (71.47 years for deceased patients and 62.49 years for hospitalized patients), allergic conditions (19.53%), and those taking other medications (46.76%) are most likely to experience side effects after vaccination, as well as those with a history of type II diabetes (3.75%), heart disease (2.08%), hypertension (4.59%), or COVID-19 infection. Also, the study found that severe reactions are mostly associated with symptoms such as hospitalization duration, headache, dyspnea (shortness of breath), fever/pyrexia, chills, fatigue/tiredness, dizziness, different kinds of pain, rash, and physical disability.

**Figure 8.**
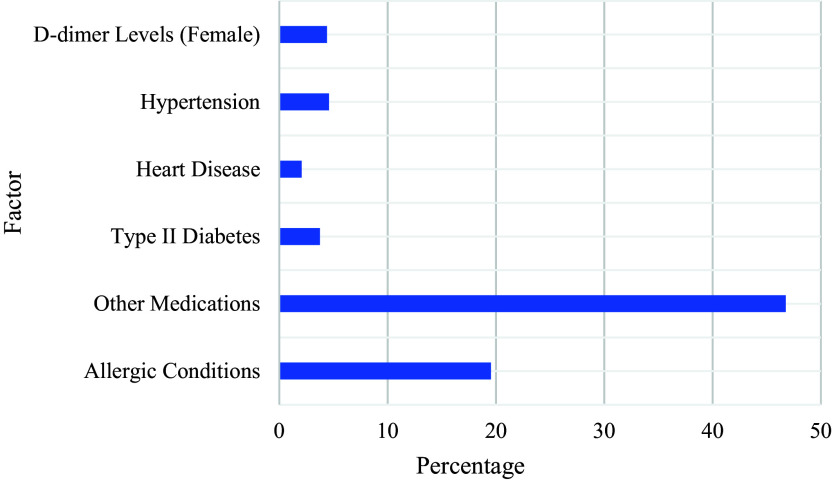
Factors associated with side effects of COVID-19 vaccines.

In addition, females were associated with higher D-dimer levels (4.4%, p-value = 0.05). In which six women who received AstraZeneca vaccines, one of whom was over 50 years old, had elevated D-dimer levels, and three developed petechiae and hematomas. one of whom was hospitalized for thromboembolism treatment ( Almufty et al. 2021). Figure 8 illustrates the various factors associated with the side effects of COVID-19 vaccines.

## Discussion

Vaccines play a vital role in reducing global rates of infections and diseases ( Lombard et al. 2007). The original assumption of vaccinations, dating back to the 11th century, was to expose a human to a small amount of disease to foster protection and immunity against exposure to a larger amount of the same pathogen. This method was 1st recorded in Chinese literature that ingesting a small amount of poison could prevent death from a larger dose of poison ( Plotkin 2005).

Vaccines induce a strong immune-response that triggers a wide range of reactions, including fever, chills, fatigue, inflammation, and swelling at the site of injection ( Marsa 2023). An “adverse event following immunization” (AEFI) is defined by the World Health Organization (WHO) as any unintended medical event that occurs after the vaccination, and which does not necessarily indicate a causal relationship with the vaccine. Symptoms, abnormal laboratory findings, or diseases that are not desirable or unintended are called adverse events ( WHO 2023).

A systematic evaluation of existing vaccination candidates was required to investigate their safety, effectiveness, immunogenicity, undesired events, and limitations. The review was conducted by scanning online resources, and 17 studies were ultimately chosen. Several types of vaccine candidates with different methods against the COVID-19 virus, including inactivated, mRNA-based, recombinant, and nanoparticle-based vaccines, are being developed and marketed, according to our findings. Depending on data from the literature, we compared these vaccines in terms of side effects. We discovered that mRNA vaccines looked to be more effective, Adenovirus Viral vector COVID-19 vaccines had a higher incidence of side effects, while inactivated vaccinations had fewer side effects.

Pain at the injection site was the most commonly reported side effect (59.1%; CI: 25.7% to 92.1%) , which conformed with the Fact sheets for post-vaccine trials ( Pfizer-BioNTech COVID-19 vaccine side effects, 2023; Moderna COVID-19 vaccine side effects, 2023; Sinopharm COVID-19 vaccine side effects, 2023; AstraZeneca COVID-19 vaccine side effects, 2023; Bharat Biotech COVID-19 vaccine side effects, 2023); localized pain has been the most common side effect in numerous other injectable vaccine trials conducted previously ( Nichol et al. 1996). In addition, tiredness (57.5%; CI: 37.7% to 86.9%), headaches (39.7%; CI: 17.8% to 90.3%), and fever (54.4%; CI: 32.6% to 69.7%) were the most commonly reported systemic side effects by the participants. In contrast, the other effects weren’t much-repeated, but it was individual states ( Figure 6D).

Although there were no serious side effects reported in most of the studies, facial paralysis and Bell’s palsy were also observed in a few cases, which resulted in life-threatening adverse reactions ( El-Shitany 2021; Ahamad 2020). Additionally, tachycardia, lymphadenopathy, and anaphylaxis were reported in a few patients ( Riad et al. 2021; Almufty et al. 2021; Dziedzic et al. 2021). Anaphylaxis (an acute allergic reaction) following vaccinations can occur due to vaccination antigens, preservatives, and stabilizers in the vaccine formulation, or residual nonhuman proteins ( Kim et al. 2021).

Among the included studies, three studies reported the incidence of oral and skin-related side effects. The most common oral side effects were vesicles (6.3%), bleeding gingiva (4.3%), oral paraesthesia (2.2%), dysgeusia (2.4%), blisters (36%), and halitosis (25.4%). While rash and urticaria (other than at the site of the injection) were the most reported skin-related side effects. There was a higher incidence of oral reactions in those who received only one vaccine dose (mRNA or viral vector-Based) and young age participants. While the prevalence was similar among males & females.

Skin-related side effects were more prevalent among females (22.6%), those who received two vaccine doses (mRNA or viral vector-Based), and participants aged ≤ 43 > 43 years. In general, the viral vector-Based vaccines were associated with the highest risk of oral and skin-related side effects, while an mRNA vaccine is associated with the lowest. Three-fourths (75.1%) of the oral side effects appeared within the first week following vaccination. The most affected sites for skin-related side effects were the upper limb, face, chest (trunk), lower limb, and back. As for oral-related side effects, the most commonly affected areas were the lips, labial, tongue, palate, and gingiva.

Most studies didn’t report delayed side effects, but Alhazmi’s study showed that delayed side effects were reported in 1% of the participants, with onset occurring at ≥ three days after vaccination ( Alhazmi et al. 2021). It was observed that, in general, these reactions began on the first day after getting a vaccination, regardless of the dosage and most of these side effects resolve within one-three days. Fewer than 1% of studies participants required hospitalization. We discovered that mRNA vaccines looked to be more effective, while inactivated vaccinations had fewer side effects. The vaccine’s effectiveness far surpasses any concerns regarding side effects.

### Gaps and risk of bias in included studies

For studies that are based on the machine learning approach: Because there was no input data to train from and the model was learning from raw data without any prior knowledge, utilizing unsupervised learning in Abdulrahman et al. (2021) resulted in a less accurate outcome. This is time-consuming because the algorithm’s learning phase, which analyzes and calculates all options, might take a long. Although supervised learning is used in studies ( Ahamad 2020; Hatmal et al. 2021) and obtains higher accuracy, it is not really considered high accuracy because they work on small datasets.

As for studies based on a statistical approach: There were many problems that were observed, the most important of which is the small sample size, as in the studies ( Jayadevan 2021; Azimi et al. 2021). The most apparent problem in studies related to the side effects of vaccines is that the number of vaccinators of one sex exceeds the other in the sample of studies ( Riad et al. 2021; Zahid 2021; Andrzejczak-Grządko et al. 2021; Kadali et al. 2021; Dziedzic et al. 2021), and this makes the results inaccurate. As is the case for the paper ( Alhazmi et al. 2021), which was limited to a certain age group, namely young people, and ignored the rest of the age groups that constitute a large part of the community. Among the other gaps that were noted is not specifying which dose led to the emergence of the offending effects, as in the paper ( Klugar et al. 2021), or relying on short-term effects in analyzing the side effects, as in the papers ( Alamer et al. 2021; Kitagawa et al. 2022). “ Table 5” below explains these studies in detail.

**Table 5.** The comparison among literature review.

Ref. No.		Author(s) &Year	Country	Study Population		Sample size	Sex Female & Male	Age (Year)	Vaccine Type	Methods	Dataset	Limitations
( Abdulrahman et al. 2021)		Abdulrahman. [May 17, 2020]	Iraq	General inhabitants		5471	N/A	N/A	Pfizer-BioNTech, Moderna vaccines	clustering algorithms (K-means, expectation-maximization, heretical, and density-based) were used with the WEKA tool.	COVID-19 World Vaccine Adverse Reactions dataset	N/A
( Ahamad 2020)		Ahmad [April 18, 2021]	Bangladesh	General inhabitants		5209	Male = 31.3% Female = 63.1 %	N/A	mRNA-based vaccines	Machine-learning classification algorithms	COVID-19 World Vaccine Adverse Reactions dataset	Small dataset, focus on a certain place and on mRNA vaccine only.
( Hatmal et al. 2021)		Hatmal [2021]	Jordan	General inhabitants		2213	Male = 39.2% Female = 60.8%	<20 >60	Sinopharm, AstraZeneca, Pfizer-BioNTech	Multilayer perceptron, extreme gradient boosting, random forest, and K-	The dataset was collected by an online survey	The sample’s gender and profession were not evenly distributed, and misclassification of results, and a self-reported online survey.
( Jayadevan 2021)		Jayadevan [February 12, 2021]	India	Healthcare Workers		5396	Male = 56% Female = 44%	≥20 ≤90	Covaxin, Pfizer BioNTech, and Sinopharm vaccine	Descriptive Statistics, Inferential Statistics. all analysis was done using SPSS software.	The dataset was collected through A cross-sectional online survey	The survey was designed in one language which may be difficult to understand for individuals with LEP, the response rate varies, making comparisons difficult.
( El-Shitany 2021)		ElShitany [April 19, 2021]	Saudi Arabia	General inhabitants		455	Male = 35.8% Female = 64.2%	<60 ≥60	Pfizer-BioNTech vaccine	Chi-square test and for data analysis Prism was used	The dataset was collected through an online survey	Small sample size, only Arab were included in the study, focusing only on the vaccine’s short-term side effects.
( Zahid 2021)		Zahid [Nov22, 2021]	Bahrain	General inhabitants		311	Male = 56.3% Female = 43.7%	≥18 ≥45	Pfizer BioNTech, Sinopharm, AstraZeneca, and Sputnik	Univariate models, the Shapiro–Wilk test, and Pearson’s correlation	The dataset was collected by A self-administered online survey	In the self-reported survey, misclassification of post-vaccination side effects resulted in information bias, sample's gender and profession were not evenly distributed.
( Andrzejczak-Grządko et al. 2021)		Andrzejczak Grządko [2021]	Poland	General inhabitants		705	Male = 15% Female = 85%	20-84	PfizerBioNTech, AstraZeneca	N/A	The dataset was collected through an online survey	due to women outnumbering men among those who have been vaccinated, the association between side effects and age, comparing females and men results can't be established
( Almufty et al. 2021)		Almufty [July 12, 2021]	Iraq	General inhabitants		1012	Male = 60.2% Female = 39.8%	≥18 ≥70	Sinopharm, AstraZeneca, Pfizer BioNTech	Descriptive Statistics, Inferential Statistics. all analysis was done using SPSS software	The dataset was collected by A standardized questionnaire platform	N/A
( Alamer et al. 2021)		Alamer [Nov 9, 2021]	Saudi Arabia	General inhabitants		965	Male = 48% Female = 52%	12–18	Pfizer- BioNTech vaccine	t-test and chi square test. all analysis was done using SPSS v.23 (IBM Corp., Armonk, NY, USA)	The dataset was collected by a self-administered online survey	focus only on vaccine short-term side effects, the survey was distributed according to the author’s networks, a self-reported online survey
( Dziedzic et al. 2021)		Dziedzic [Nov16, 2021]	Poland	Healthcare Workers and medical student		247	Male = 20.2% Female = 79.8%	≤29 >29	Pfizer-BioNTech, Moderna, AstraZeneca	Descriptive Statistics, Inferential Statistics. all analysis was done using SPSS v.27.0 software	The dataset was collected by an internet-based survey platform called Survey Monkey.	The self-reported survey had, a moderate sample size that impacted the results; the sample and its demographic characteristics were neither evenly distributed nor equivalent to the population profile, partial-non-response bias.
( Azimi et al. 2021)		Azimi [Oct 2, 2021]	Afghanistan	Healthcare Workers		400	Male = 81.3% Female = 18.8%	≤40 >40	AstraZeneca	Descriptive Statistics and chi-square test. All analysis was done using IBM SPSS v.25 software	The dataset was collected through an online survey	Limited information about second dose side effects intensity, the small sample size that affects the result generalization
( Klugar et al. 2021)		Klugar [Aug 5, 2021]	Czech Republic		Healthcare Workers	599	Male = 27.2% Female = 72.3%	≤39 ≥39	Pfizer BioNTech, Moderna, AstraZeneca	Descriptive Statistics, Inferential Statistics. all analysis was done using SPSS v.27.0 software	The dataset was collected by self-administered questionnaire by KoBoToolbox for data collection	the response rate varies, making comparisons between doses difficult; it wasn't determined after which of the doses the side effects occurred; using KoBo Toolbox software made it difficult to know the response rate, and =the sample size was not optimal due to the limited number of HCWs who receive Moderna, AstraZeneca vaccines.
( Riad et al. 2021)		Riad [April 1, 2021]	Czech republic		Healthcare Workers	877	Male =11.4% Female = 88.5%	≤43 ≥43	Pfizer-BioNTech	Descriptive Statistics, Inferential Statistics. all analysis was done using SPSS v.27.0 software	The dataset was collected by self-administered questionnaire by KoBoToolbox for data collection	Due to the self-reported online survey, self-selection bias could occur, the sample's gender and profession were not evenly distributed, and it wasn't determined which of the doses caused side effects.
( Kitagawa et al., 2022)		Kitagawa [11Jan,2022]	Japan		Healthcare Workers	12214	Male = 38.2% Female = 61.8%	<20 ≤79	Pfizer-BioNTech, Moderna	Inferential Statistics. all analysis was done using JMP Pro 16.0 software	The dataset was collected by self-reported online survey	the number of side effects reported was exaggerated or underestimated, only short-term side effects were investigated, and their intensity and duration were not analyzed, due to an unidentified vaccination date, recall bias exists, participants' medical history was not investigated, no distinction was made between HCWs and non-HCWs, anaphylaxis or myocarditis symptoms were not included
( Alhazmi et al. 2021)		Alhazmi [June 18, 2021]	Saudi Arabia		General inhabitants	515	Male = 43% Female = 57%	18 – 70	Pfizer BioNTech, AstraZeneca vaccines	Descriptive Statistics, Inferential Statistics, and multivariate logistic regression models. All analysis was done using SPSS v.23 software	The dataset was collected through an online survey	The majority of the participants were young, the self-reported survey may result in reporting bias, and the survey was distributed according to the author’s networks.
( Saeed et al. 2021)		Saeed [August 9, 2021]	UAE		General inhabitants	1080	Male = 29.6% Female = 70.4%	≥18 ≤80	Sinopharm vaccine	Descriptive Statistics, Inferential Statistics. all analysis was done using SPSS v.22.0 software	The dataset was collected by self-reported online survey	N/A
( Kadali et al. 2021)		Kadali [April 15, 2021]	USA		Healthcare Workers	1116	Male =10.42% Female = 89%	≥18 ≤80	Moderna vaccine	N/A	The dataset was collected by an online survey through an internet-based survey platform called Survey Monkey.	Since it was a web-based survey, the vaccine receiving and the reported side effects were not verified., The time of occurrence and duration of post-vaccination side effects were not specified and 89.35% of females in their study so there may be a risk of bias

### Strengths and limitations

As far as the authors are aware, this is the first study to analyze various kinds of side effects of COVID-19 vaccines. Furthermore, this study pointed out possible factors that might contribute to these effects.

This study has some limitations. Despite not restricting language in the search process, we used only English-language databases, so publications in other languages have been missed, leading to publication bias. Studies did not provide information about certain groups, such as pregnant women. Furthermore, some of the participants in the analyzed studies may have reported side effects in a biased way. This could be related to the disparity in their educational levels. Therefore, the reliability of reported side effects may be affected by reporting bias in the included studies.

## Conclusion and future works

In conclusion, this scoping systematic review investigated the side effects associated with COVID-19 approved vaccines including Pfizer BioNTech (Comirnaty), Moderna (mRNA-1273), Sinopharm (BBIP CorV), and AstraZeneca (ChAdOx1), as well as Bharat Biotech (Covaxin). Most of the side effects were mild, self-limiting, and common. Thus, they usually resolve within 1–3 days after vaccination. Currently, all types of vaccines that are approved still outweigh the possible risks of these vaccines, and the vaccinations are strongly recommended when it is available.

In future works, it is necessary to study the side effects more accurately, and that is done through the correct selection of the study community, which begins with the selection of the study population. The study sample must ensure appropriate representation of men and women, as gender bias was a major loophole in the studies we adopted and had a significant impact on the real results obtained.

### Reporting guidelines

Figshare: PRISMA-P checklist for ‘COVID-19 vaccinations and their side effects: A scoping systematic review’. https://doi.org/10.6084/m9.figshare.22722592.

Data are available under the terms of the Creative Commons Attribution 4.0 International license (CC-BY 4.0).

## Data Availability

All data supporting the results are available as part of the article and no additional source data are required.
