# Supplementary material for: COVID-19 vaccinations and their side effects: a scoping systematic review
Source: F1000Res. 2024 Sep 26;12:604. Originally published 2023 Jun 5. [Version 2] doi: 10.12688/f1000research.134171.2 (PMC11541072; doi:10.12688/f1000research.134171.2)
Supplement: Peer review history

# Reviewer report: Reviewer response for version 2

**DOI:** 10.5256/f1000research.172135.r327102

**Published:** 2024-11-06

**Authors:** Bekir Kocazeybek

**Affiliations:** 1 Istanbul University—Cerrahpasa, Istanbul, Turkey

Revisions are satisfactory.

Are the rationale for, and objectives of, the Systematic Review clearly stated?

Partly

Is the statistical analysis and its interpretation appropriate?

Yes

If this is a Living Systematic Review, is the ‘living’ method appropriate and is the search schedule clearly defined and justified? (‘Living Systematic Review’ or a variation of this term should be included in the title.)

Partly

Are sufficient details of the methods and analysis provided to allow replication by others?

Partly

Are the conclusions drawn adequately supported by the results presented in the review?

Yes

Reviewer Expertise:

Microbiology, immunology

I confirm that I have read this submission and believe that I have an appropriate level of expertise to confirm that it is of an acceptable scientific standard.

# Reviewer report: Reviewer response for version 1

**DOI:** 10.5256/f1000research.147203.r309618

**Published:** 2024-08-23

**Authors:** Bekir Kocazeybek

**Affiliations:** 1 Istanbul University—Cerrahpasa, Istanbul, Turkey

COVID-19 VACCINATIONS AND THEIR SIDE EFFECTS: A SCOPING SYSTEMATIC REVIEW

 It was evaluated according to the PRISMA 2020 Checklist. The article needs major revision before indexing. My recommendations:

 Is the Grandini et al.2020 study included in the 17 selected studies? If so, it is not included in Table 5, where all studies are shown.

 It is mentioned as if Hatmal et al.2021 was included in the study. Although it has an explanation and evaluation, its name is not in Table 5 and the list of studies.

 It is recommended that notifications be made in accordance with PRISMA 8- “Indicate the methods used to decide whether a study meets the criteria for inclusion in the review, including how many reviewers screened each record and whether each report was worked on independently and, if appropriate, details of the automation tools used in the process.”

 It is recommended to make notifications in accordance with PRISMA 9- “Specify the methods used to collect data from reports, how many reviewers collected data from each report, whether they worked independently, the process for obtaining or approving data from study investigators, and details of automation tools used in the process, if any.”

 It is recommended to add a study in accordance with PRISMA 16-b “Cite studies that may appear to meet the inclusion criteria but were excluded and explain why they were excluded.”

 PRISMA 17- “Cite each included study and provide its characteristics.” Ma’mon,2021 is mentioned in the study but its description is not included in the Review of the conducted studies section.

 It is recommended to make notifications in accordance with PRISMA 18- “Provide assessments of risk of bias for each included study.”

 It is recommended to make notifications in accordance with PRISMA 22- “Provide assessments of precision (or reliability) as evidence for each outcome assessed.”

Are the rationale for, and objectives of, the Systematic Review clearly stated?

Partly

Is the statistical analysis and its interpretation appropriate?

Yes

If this is a Living Systematic Review, is the ‘living’ method appropriate and is the search schedule clearly defined and justified? (‘Living Systematic Review’ or a variation of this term should be included in the title.)

Partly

Are sufficient details of the methods and analysis provided to allow replication by others?

Partly

Are the conclusions drawn adequately supported by the results presented in the review?

Yes

Reviewer Expertise:

Microbiology, immunology

I confirm that I have read this submission and believe that I have an appropriate level of expertise to state that I do not consider it to be of an acceptable scientific standard, for reasons outlined above.

# Response

**Published:** 2024-09-18

**Authors:** Hind Monadhel

**Affiliations:** Computer Science, University of Technology Baghdad, Baghdad, Baghdad, Iraq

Dear Dr. Bekir Kocazeybek,

 Thank you for your thoughtful and detailed comments. I appreciate the opportunity to clarify and address the points you raised.

 Regarding Grandini et al. (2020):

 I would like to clarify that Grandini et al. (2020) is cited as a reference for the equation used in the analysis but is not included among the 17 studies reviewed. Therefore, it is not listed in Table 5. I appreciate your attention to this detail, and I hope this resolves any confusion.

 Regarding Hatmal et al. (2021):

 You are correct that Hatmal et al. (2021) is mentioned in the manuscript. This study is included among the 17 studies in Table 5, but it was mistakenly cited under the name M. Ma'mon et al. I have now corrected this citation discrepancy to ensure consistency throughout the document. I hope this resolves the concern.

 PRISMA 18 - Risk of Bias:

 The recommendation to assess the risk of bias for each included study has already been addressed in the "Gaps in Conducted Studies" section, where I discuss limitations in study design, sample size, and methodological weaknesses that indicate potential bias.

 PRISMA 22 - Assessments of Precision:

 In line with PRISMA 22, I have now included assessments of precision (or reliability) for some outcome, which can be found in the discussion section of the manuscript.

 Once again, I sincerely appreciate your valuable feedback and your effort to ensure the rigor of the manuscript. I hope these revisions address your concerns, and I look forward to any further comments  you may have.

# Reviewer report: Reviewer response for version 1

**DOI:** 10.5256/f1000research.147203.r218626

**Published:** 2023-11-24

**Authors:** Yongbin Wang

**Affiliations:** 1 The First Affiliated Hospital, Xinxiang Medical University, Xinxiang, Henan Province, China

This manuscript performs a scoping systematic review to explore the COVID-19 vaccinations and their side effects, this topic is very interesting and has important guiding significance for evaluating the effectiveness of vaccines. Although this paper did a good job, the current version needs to be further improved by a minor revision.

**Title:**

 The title is concise and summarizes the content of the article.

**Abstract:**

 The abstract is concise and effectively outlines the research's objectives, which include evaluating the COVID-19 vaccinations and their side effects. However, several minor points need to be revised:

1. Results need to provide figures to support the findings instead of something like a conclusion.
2. Conclusion should give a summary of adverse reactions of the vaccine.

**Introduction:**

 The introduction provides adequate context for understanding the research problem and the primary hypotheses. Also, some minor points need to be revised:

1. Paragraph 1 should summarize the severity of the impact of COVID-19, including the number of cases and deaths.
2. Paragraph 4 should provide the recent figures regarding the vaccine doses, this data is available on the WHO website.
3. It should be highlighted that the duration of application of COVID-19 vaccines is short, and adverse effects are inconsistent due to different types of vaccines.

**Background:**

 This part is well-written, particularly the method used in this study, giving an adequate description. Here just needs a very minor revision:

1. “A brief overview of the vaccine” should be removed.
2. Check **"**from 2019 to 2022 to select eligible research publications."

**Results:**

 The results are also adequate. Factors associated with side effects of COVID-19 vaccines can provide a summary in Table or Figure.

**Discussion:**

 The discussion also gives a full comparison with other studies. But some minor points should be addressed:

1. In the discussion, can the authors explain the causes of adverse reactions of different vaccines from the mechanism level?
2. Some new research results have been published recently, and the discussion needs to be supplemented and fully compared.

Are the rationale for, and objectives of, the Systematic Review clearly stated?

Yes

Is the statistical analysis and its interpretation appropriate?

Yes

Are sufficient details of the methods and analysis provided to allow replication by others?

Yes

Are the conclusions drawn adequately supported by the results presented in the review?

Yes

Reviewer Expertise:

NA

I confirm that I have read this submission and believe that I have an appropriate level of expertise to confirm that it is of an acceptable scientific standard.

# Response

**Published:** 2024-09-18

**Authors:** Hind Monadhel

**Affiliations:** Computer Science, University of Technology Baghdad, Baghdad, Baghdad, Iraq

Dear Dr. Wang,

 Thank you for your thoughtful and constructive feedback on our manuscript. We have carefully considered your comments and made the necessary revisions to improve the paper. We sincerely appreciate your insightful suggestions, which have significantly contributed to strengthening the manuscript
